# Biological correlates of complex posttraumatic stress disorder—state of research and future directions

**DOI:** 10.3402/ejpt.v6.25913

**Published:** 2015-04-16

**Authors:** Zoya Marinova, Andreas Maercker

**Affiliations:** 1 Department of Psychology, Division of Psychopathology and Clinical Intervention, University of Zürich, Zürich, Switzerland; 2 University Clinics for Child and Adolescent Psychiatry, University of Zürich, Zürich, Switzerland

**Keywords:** Complex posttraumatic stress disorder, posttraumatic stress disorder, biological correlate, complex trauma, pathophysiology

## Abstract

Complex posttraumatic stress disorder (PTSD) presents with clinical features of full or partial PTSD (re-experiencing a traumatic event, avoiding reminders of the event, and a state of hyperarousal) together with symptoms from three additional clusters (problems in emotional regulation, negative self-concept, and problems in interpersonal relations). Complex PTSD is proposed as a new diagnostic entity in ICD-11 and typically occurs after prolonged and complex trauma. Here we shortly review current knowledge regarding the biological correlates of complex PTSD and compare it to the relevant findings in PTSD. Recent studies provide support to the validity of complex PTSD as a separate diagnostic entity; however, data regarding the biological basis of the disorder are still very limited at this time. Further studies focused on complex PTSD biological correlates and replication of the initial findings are needed, including neuroimaging, neurobiochemical, genetic, and epigenetic investigations. Identification of altered biological pathways in complex PTSD may be critical to further understand the pathophysiology and optimize treatment strategies.

Posttraumatic stress disorder (PTSD) is a mental disorder, which occurs in response to a severe traumatic event and includes several core symptoms: re-experiencing the event accompanied by intense distress, persistently avoiding reminders of the traumatic event, and a state of hyperarousal. PTSD is associated with significant impairment of social as well as occupational functioning, which presents a severe burden for the affected individuals and society (Kessler, 2000). Furthermore, treatment success is often limited, leading to a protracted or recurrent course of symptoms appearing or recurring years after the traumatic event.

The concept of partial (subthreshold) PTSD has long been discussed in order to provide diagnostic coverage for individuals suffering from PTSD symptoms, but not meeting full PTSD diagnostic criteria (Schützwohl & Maercker, 1999; Stein, Walker, et al., 1997). Functional impairment degree and comorbidity rates in partial PTSD have been found to be intermediate between those of traumatized subjects without PTSD symptoms or with full PTSD (McLaughlin et al., 2015). Partial PTSD has been associated with increased risk for mood and anxiety disorders, substance use, suicide attempts, and medical comorbidities in comparison to traumatized individuals without PTSD symptoms (Pietrzak, Goldstein, Southwick, & Grant, 2011, 2012).

## Complex PTSD—symptoms and its proposed inclusion as a new diagnostic entity in the ICD-11

Exposure to multiple traumatic events, especially in childhood, has been associated with a more complex trauma symptomatology later in life (Briere, Kaltman, & Green, 2008; Cloitre et al., 2009). It was conceptualized in 1992 by Herman that after prolonged traumatic experience, complex PTSD may occur (Herman, 1992). Complex PTSD has been proposed for inclusion as a new diagnostic entity in the ICD-11 by the working group for “disorders specifically associated with stress” of the World Health Organization (Maercker et al., 2013a, 2013b). The planned complex PTSD category will include three additional clusters of symptoms besides the core PTSD symptoms: persistent and pervasive problems in emotional regulation (increased emotional reactivity, lack of affect, violent outbursts), negative self-concept (feelings of defeat, worthlessness, guilt, or shame), and problems in interpersonal relations (difficulties in establishing or maintaining relationships with others). Complex PTSD will be diagnosed when symptoms from these three additional domains are present in addition to the core symptoms of full or partial PTSD (Table 1). It is important to note that the definition of complex PTSD will be based on the disease symptomatology and not on the specific trauma. However, complex PTSD is most commonly associated with traumatic events of prolonged nature that the victim could not escape. Trauma types associated with complex PTSD include childhood maltreatment, torture, imprisonment, war and refugee status, or long-lasting interpersonal violence (Bryant, 2010).

**Table 1:** Symptom domains related to PTSD and complex PTSD according to the proposed ICD-11 diagnostic criteria

PTSD	Complex PTSD
Re-experiencing a traumatic event	Re-experiencing a traumatic event
Avoiding reminders of the event	Avoiding reminders of the event
Hyperarousal	Hyperarousal
	Affect dysregulation
	Negative self-concept
	Problems in interpersonal relationships

Multiple diagnostic iterations of complex PTSD-related symptoms have been proposed. Some features related to complex PTSD symptomatology have been encompassed by “Disorders of Extreme Stress Not Otherwise Specified” (DESNOS) (Pelcovitz et al., 1997); “PTSD and Its Associated Features” in the Diagnostic and Statistical Manual of Mental Disorders, Fourth Edition (DSM-IV) (APA, 2000); and “Enduring Personality Change after Catastrophic Events” (EPCACE) in the ICD-10 (WHO, 1992). In addition, the term “Developmental Trauma Disorder” has been used to describe the clinical presentation in children and adolescents subjected to complex trauma (Van der Kolk, 2005).

An alternative approach is implemented in DSM-5, in which symptoms related to the complex PTSD concept are considered a part of the core symptom profile of PTSD. This is accomplished through introducing a new cluster D of PTSD symptoms, which concern negative mood and cognition changes associated with the traumatic event (including among others negative self-beliefs, feeling of detachment from others, and negative emotional states) (APA, 2013). DSM-5 also includes a dissociative PTSD subtype to diagnose individuals who in addition to the core PTSD symptoms demonstrate significant dissociative symptoms, including depersonalization and/or derealization. Although the proposed for ICD-11 complex PTSD diagnostic criteria do not particularly emphasize dissociation, the DESNOS concept considered dissociative symptoms besides other symptom domains.

We review studies focusing on validating the diagnostic concept and identifying biological correlates of complex PTSD. Key biological correlates of PTSD are pointed out to contextualize complex PTSD findings and to outline potential areas for complex PTSD research. Finally, we present future research directions for the identification of complex PTSD neurobiological correlates.

## Search strategy

Relevant studies were identified by searching the database PUBMED (http://www.ncbi.nlm.nih.gov/pubmed/) for the terms “complex posttraumatic stress disorder” OR “complex PTSD” OR “disorders of extreme stress not otherwise specified” OR “SIDES.” Original articles, including the search terms and published through 28 February 2015, were analyzed to select those identifying complex PTSD biological correlates or assessing its validity as a diagnostic concept. In addition, we searched for and separately analyzed original articles including the terms “childhood sexual abuse” AND “PTSD” OR “posttraumatic stress disorder.” PTSD-affected individuals with history of childhood sexual abuse (CSA) are expected to present with especially high rates of complex PTSD symptoms (McLean & Gallop, 2003; Roth, Newman, Pelcovitz, Van der Kolk, & Mandel, 1997; Zlotnick et al., 1996).

## Validating the diagnostic concept of complex PTSD

Several recent studies have assessed the validity of the proposed for ICD-11 complex PTSD as a diagnosis distinct from PTSD. A study by Cloitre, Garvert, Brewin, Bryant, and Maercker (2013) demonstrated the presence of three trauma victims’ classes using latent profile analysis (LPA)—scoring high only on PTSD symptoms, high on complex PTSD symptoms, or low on all PTSD and complex PTSD symptoms. In this investigation, complex PTSD symptomatology was more often associated with chronic trauma and more highly predictive of impairment. Complex PTSD construct validity was also assessed in a group of adults subjected to childhood institutional abuse (Knefel, Garvert, Cloitre, & Lueger-Schuster, 2015; Knefel & Lueger-Schuster, 2013), in which complex PTSD was indicated as a relevant diagnosis by a strong model fit using confirmatory factor analysis and LPA. In addition, latent class analysis in three distinct trauma groups (victims of sexual assault, physical assault, or bereavement) provided additional evidence for the fit of complex PTSD as a separate class and its association with serious psychological problems (Elklit, Hyland, & Shevlin, 2014). A recent study from our group further supported the validity of complex PTSD as a separate diagnostic entity in a German community sample of 3,012 adolescents and young adults subjected to interpersonal violence. LPA in this sample supported a four-class fit, in which individuals with high degree of PTSD symptoms and individuals with high degree of complex PTSD symptoms were presenting as two distinct classes (Perkonigg et al., submitted). Altogether, these studies underscore the validity of the diagnostic concept of complex PTSD and the association of complex PTSD symptoms with additional psychological problems, potentially indicative of higher impairment.

On the other hand, the diagnostic utility of the dissociative PTSD subtype has also been supported by studies utilizing LPA (Steuwe, Lanius, & Frewen, 2012; Wolf et al., 2012).

## Neuroimaging studies and complex PTSD

In adult PTSD patients, reduced hippocampal, left amygdala, and anterior cingulate cortex (ACC) volumes have been detected (Bremner et al., 1995; meta-analysis by Karl et al., 2006). A recent meta-analysis by Meng et al. (2014) corroborated these findings, identifying volume reduction of the left ACC, the left insula, and the right parahippocampal gyrus. Functional brain changes in the hippocampus, amygdala, and prefrontal cortex have also been found in PTSD patients (Bremner, 2007a). A meta-analysis, summarizing functional MRI (fMRI) and positron emission tomography studies on PTSD, confirmed mid- and dorsal ACC and amygdala hyperactivaty, as well as ventromedial prefrontal cortex and inferior frontal gyrus hypoactivity (Hayes, Hayes, & Mikedis, 2012).

Several imaging studies have been conducted on complex PTSD subjects. In childhood abuse-related complex PTSD, decreased gray matter concentration in the right hippocampus, the right dorsal ACC, and the right orbitofrontal cortex (OFC) was observed (Thomaes et al., 2010). These findings imply that complex PTSD may be associated with more severe neural imaging correlates than PTSD. An fMRI study on childhood abuse-related complex PTSD indicated altered activation of the left hippocampus and parahippocampal gyrus, suggesting hippocampal dysfunction (Thomaes et al., 2009). Furthermore, in complex PTSD disturbances in the activation of the left ventral ACC and dorsal ACC, the dorsomedial prefrontal cortex, left ventrolateral prefrontal, and OFC were observed, implying involvement of regions particularly important for emotional processing (Thomaes et al., 2013). Reversal after psycho-educational and cognitive behavioral stabilizing group treatment of some of the neuroimaging alterations in complex PTSD patients was also detected (Thomaes et al., 2012). All four studies assessing neuroimaging correlates associated with complex PTSD (Table 2) focused on childhood abuse-related complex PTSD. Their replication by independent investigations of complex PTSD related to other trauma types is warranted. In addition, these studies included probands with PTSD symptoms and complex PTSD symptoms consistent with the DESNOS conceptualization. Thus, they do not directly support the proposed by the ICD-11 complex PTSD criteria, but rather the broader concept of biological correlates associated with complex PTSD-related symptoms.

**Table 2:** Studies on biological correlates of complex PTSD

Authors	Sample	Diagnostic tool	Method	Key findings
Thomaes et al., 2009	Child abuse-related complex PTSD	CAPS SIDES	fMRI	↑ activation in the left hippocampus and parahippocampal gyrus during preferential recall of negative words
Thomaes et al., 2010	Child abuse-related complex PTSD	CAPS SIDES	Structural MRI	↓ hippocampal volume ↓ ACC volume ↓ right orbitofrontal cortex volume
Thomaes et al., 2012	Child abuse-related complex PTSD	CAPS SIDES	fMRI	trend for ↑ left anterior insula and dorsal ACC activation in the classic Stroop task after therapy ↓ dorsal ACC and left anterior insula activation
Thomaes et al., 2013	Child abuse-related complex PTSD	CAPS SIDES	fMRI	(during encoding of negative words) ↑ response in the left ventral ACC and dorsal ACC extending to the dorsomedial prefrontal cortex trend for ↑ left hippocampus activation

fMRI=functional magnetic resonance imaging; complex PTSD=complex posttraumatic stress disorder; ACC=anterior cingulate cortex.

The existence of a dissociative PTSD subtype has also been suggested by imaging studies showing distinct patterns of brain activation in patients presenting with PTSD with or without dissociative symptoms (Lanius, Brand, Vermetten, Frewen, & Spiegel, 2012).

## Areas of interest for future complex PTSD research based on previous PTSD findings

### Autonomic and neurochemical measures

Hyperactivity of the sympathetic branch of the autonomic nervous system has been observed in PTSD patients, including higher blood pressure, heart rate, and increased skin conductance (reviewed in Sherin & Nemeroff, 2011). In addition, PTSD patients show exaggerated response to traumatic reminders, associated with increases in heart rate, blood pressure, and norepinephrine levels.

Norepinephrine and serotonin signaling dysregulation has repeatedly been implicated in PTSD (Krystal & Neumeister, 2009). Alterations in the signaling of amino acids (γ-aminobutyric acid—GABA and glutamate), peptides (neuropeptide Y and endogenous opioid peptides), and thyroid hormones have also been associated with PTSD (Sherin & Nemeroff, 2011). PTSD has been correlated with hypocortisolism, although not all studies have replicated this phenomenon. A meta-analysis suggested the measurement method, sex, and trauma type could influence hypocortisolism detection in PTSD (Meewisse, Reitsma, De Vries, Gersons, & Olff, 2007).

Investigation of the overlap and potential differences in autonomic and neurobiochemical alterations associated with PTSD and complex PTSD is an important research avenue, which can help to understand the pathophysiology of the disorder and may even contribute to novel therapeutic targets identification.

### Genetic predisposition

PTSD symptoms heritability between 30 and 40% has been estimated; however, there has been a relatively small number of genetic variants identified in PTSD patients (Cornelis, Nugent, Amstadter, & Koenen, 2010; Stein, Jang, Taylor, Vernon, & Livesley, 2002; True et al., 1993). Most studies have focused on polymorphisms in genes associated with the neurotransmitters dopamine, serotonin, and norepinephrine; genes related to the hypothalamic-pituitary-adrenal axis (HPA axis); and neurotrophic factors (reviewed in Cornelis et al., 2010). In addition, a single nucleotide polymorphism (SNP) in the retinoid-related orphan receptor alpha gene, implicated in neuroprotection, was identified by a genome-wide association study (GWAS) as a PTSD associated variant (Logue et al., 2013).

So far no studies have specifically investigated the role of genetic predisposition for complex PTSD. Such information would provide insight into the heritability range of complex PTSD and the extent of overlap in biological pathways involved in the genetic predisposition to PTSD and complex PTSD.

In regards to a dissociative PTSD subtype, a recent GWAS study by Wolf et al. (2014) identified in a trauma-exposed sample for 10 SNPs, a suggestive association with dissociative symptoms, which did not reach genome-wide significance.

Because genetic factors only partially account for PTSD vulnerability, the involvement of epigenetic factors in the pathophysiology of PTSD has also been hypothesized.

### Epigenetic factors

Epigenetic factors reflect the effect of environmental influences on disease occurrence and progression. Recent studies have investigated the role of epigenetic modifications, including DNA methylation, in PTSD. In particular, altered DNA methylation patterns of genes associated with immune function have been identified in PTSD (Rusiecki et al., 2013; Smith et al., 2011; Uddin et al., 2010). Changes in DNA methylation of repetitive elements have also been described (Rusiecki et al., 2012).

So far epigenetic mechanisms have not been investigated specifically in complex PTSD. Assessment of epigenetic patterns in complex PTSD would be of particular importance due to their potential reversibility, rendering them an attractive therapeutic target. A pioneering study by Yehuda et al. (2013) demonstrated an association between DNA methylation and psychotherapy-induced changes in PTSD symptoms. It showed association of *glucocorticoid receptor* and *FK506 binding protein 5 (FKBP5)* DNA methylation levels with prognosis and symptoms’ severity respectively after psychotherapy in a group of PTSD-affected combat veterans (Yehuda et al., 2013).

Interestingly, an interaction of *FKBP5* genetic variants and childhood abuse for PTSD vulnerability and symptoms severity has been demonstrated (Binder et al., 2008; Xie et al., 2010). Furthermore, for one of the *FKBP5* genetic variants, a mediating effect of DNA methylation levels for PTSD vulnerability caused by genetic polymorphism and childhood abuse interaction has been identified (Klengel et al., 2013). Such models of gene×environmental interaction and mediating effects of epigenetic factors may be highly relevant for complex PTSD vulnerability and will need to be elucidated by further studies.

### Telomere shortening

Human telomeres are repetitive sequences of nucleotides at the ends of chromosomes, which protect them from damage and foster their stability. Recent studies have demonstrated that shortened telomere length may be associated with PTSD (O'Donovan et al., 2011; Zhang et al., 2014). Although childhood trauma has also been associated with telomere shortening, the importance of the presence of childhood trauma for decrease in telomere length in PTSD patients has yet to be determined. Telomere length in complex PTSD has not been specifically investigated so far. Interestingly, epigenetic alterations or changes in telomere length may also show transgenerational effects (Küffer, Maercker, & Burri, 2014; Yehuda & Bierer, 2008).

Some major biological findings relevant to PTSD and pilot data concerning complex PTSD are summarized in Table 3.

**Table 3:** Neurobiological correlates associated with PTSD and complex PTSD

	PTSD	Complex PTSD (pilot data in need of replication)
Structural imaging studies	↓ hippocampal volume ↓ ACC volume	↓ hippocampal volume ↓ ACC volume ↓ right OFC volume
Functional imaging studies (implicated areas)	hippocampus and parahippocampal gyrus amygdala prefrontal cortex mid- and dorsal ACC amygdala ventromedial prefrontal cortex inferior frontal gyrus	hippocampus and parahippocampal gyrus ventral and dorsal ACC dorsomedial prefrontal cortex ventrolateral prefrontal cortex orbitofrontal cortex
Autonomic and Neurochemical measures	heart rate blood pressure skin conductance norepinephrine serotonin γ-aminobutyric acid (GABA) glutamate neuropeptide Y endogenous opioid peptides cortisol	
Genetic predisposition	polymorphisms in: dopaminergic, serotonergic and norepinephrinergic genes genes related to the HPA axis neurotrophic factors the retinoid-related orphan receptor alpha gene	
Epigenetic factors	DNA methylation of genes associated with immune functions DNA methylation of repetitive elements	

Studies assessing complex PTSD reviewed in the table included individuals with PTSD symptoms and complex PTSD symptoms consistent with the DESNOS conceptualization.

## Timing of the traumatic event, complex trauma, and complex PTSD symptomatology

### Developmental stage during which the traumatic event occurs

The role of the developmental stage, during which a traumatic insult occurs, for complex PTSD vulnerability and for the involvement of specific biological pathways is not completely clarified. Protracted childhood trauma has been associated with increased risk for affect dysregulation, disorganization of the self-concept, and interpersonal relationship problems; which are symptoms of complex PTSD (Briere & Rickards, 2007). Neuroimaging findings related to PTSD in abused children and adolescents have included decreased cerebral and intracranial volumes in comparison to controls, and correlation of brain volumes with abuse duration and trauma onset (De Bellis et al., 1999).

However, complex PTSD also can be observed after adulthood trauma (for example in victims of torture) even in the absence of childhood abuse (McDonnell, Robjant, & Katona, 2013). Thus, symptoms characteristic of complex PTSD were evident in ex-prisoners of war assessed 30 years after their release from captivity (Zerach & Solomon, 2013), and in female veterans who suffered military sexual assault (Luterek, Bittinger, & Simpson, 2011).

A recent study by Mehta et al. (2013) found almost completely non-overlapping genome-wide DNA methylation profiles between PTSD subjects who suffered childhood maltreatment or only adulthood trauma. The role of DNA methylation for PTSD vulnerability appeared to be greater in the childhood maltreated subjects. DNA methylation alterations in genes associated with cell survival, development, adhesion, migration, and immune function were observed in both groups. Unique pathways were related to central nervous system development and tolerance induction (in childhood maltreated PTSD subjects) and growth and apoptosis (in PTSD subjects who had not suffered childhood maltreatment) (Mehta et al., 2013). It is not clear whether these differences are due to the timing of the traumatic event or whether childhood trauma was more commonly related to a complex PTSD phenotype, contributing to the differing DNA methylation patterns.

An important focus of further investigations assessing biological correlates of complex PTSD will be to determine the overlap and differences between pathways involved in complex PTSD symptomatology after traumatic events occurring in childhood or adulthood.

### Potential insight from childhood sexual abuse-related PTSD studies

Due to the limited number of studies focusing on biological correlates of complex PTSD, we also searched for studies assessing PTSD related to CSA. Clear distinction should be made between childhood abuse-related PTSD (including CSA) and complex PTSD, because childhood abuse victims may also develop PTSD without complex PTSD symptoms. However, especially high rates of complex PTSD symptoms in CSA survivors have repeatedly been found. Thus, Zlotnick et al. (1996) established that CSA history was associated with a set of symptoms characteristic of DESNOS. In a study by Roth et al. (1997), 76% of sexually abused individuals with a lifetime diagnosis of PTSD also had the diagnosis of complex PTSD. McLean and Gallop (2003) detected complex PTSD symptoms and borderline personality disorder in 94.7% of a group of women with history of early onset sexual abuse.

Studies focusing on CSA-related PTSD have identified changes in diverse neurobiological domains in comparison to abused individuals without PTSD. These include alterations in neuroimaging, autonomic, and neurobiochemical correlates (summarized in Table 4). CSA-related PTSD studies may provide some insight in biological pathways of interest to focus on in complex PTSD patients. For example, a recent study examined the role of endocannabinoids (anandamide and 2-arachidonoylglycerol) and of fatty acid ethanolamides (related to endocannabinoids) in a PTSD sample after CSA (Schaefer et al., 2014). Serum levels of the fatty acid ethanolamide oleoylethanolamide were elevated in the CSA-related PTSD group, supporting previous genetic and imaging studies, implying a role of the cannabinoid system in PTSD.

**Table 4:** Studies on biological correlates of PTSD related to childhood sexual abuse

Authors	Sample	Key findings
Schaefer et al., 2014	CSA-related PTSD	↑ serum levels of the fatty acid ethanolamide oleoylethanolamide
Bremner, Vermetten, & Kelley, 2007b	CSA-related PTSD	resting afternoon hypocortisolemia with ↑ cortisol pulsatility
Bremner et al., 2005	CSA-related PTSD	↑ left amygdala activation with fear acquisition, and ↓ anterior cingulate function with fear extinction
Friedman, Wang, Jalowiec, McHugo, & McDonagh-Coyle, 2005	CSA-related PTSD	↑ in total T3 levels, total T3/free thyroxine ratio, free T3/total T3 ratio, and small ↓ in TSH
Klumpers et al., 2004	CSA-related PTSD	no significant differences in cardiovascular or hormonal response to standardized stress tests
Bremner et al., 2004	CSA-related PTSD	indications for a dysfunction of a network of brain regions including the visual and parietal cortex and the anterior cingulate
Bremner et al., 2003b	CSA-related PTSD	indications for a dysfunction of a network of brain regions including the medial prefrontal cortex, hippocampus and cingulate in a study of emotionally valenced declarative memory
Bremner et al., 2003a	CSA-related PTSD	↓ hippocampal volume and failure of hippocampal activation during verbal declarative memory tasks
Wilson, Van der Kolk, Burbridge, Fisler, & Kradin, 1999	CSA-related PTSD	↑ index of lymphocyte activation (CD45RO/CD45RA)
Shin et al., 1999	CSA-related PTSD	↑ orbitofrontal cortex and anterior temporal pole and ↓ increases in anterior cingulate gyrus blood flow compared to non-PTSD traumatized group
Bremner et al., 1999	CSA-related PTSD	dysfunction of the medial prefrontal cortex, visual association cortex and hippocampus in pathological memories of childhood abuse ↑ activation in motor cortex and posterior cingulate
Orr et al., 1998	CSA-related PTSD	larger physiologic responses during abuse-related scripts
Stein, Yehuda, et al., 1997	CSA-related PTSD	↑ suppression of plasma cortisol in response to dexamethasone
Lemieux & Coe, 1995	CSA-related PTSD	sympathetic and adrenocortical activation

CSA=childhood sexual abuse; PTSD=posttraumatic stress disorder.

## Significance of complex PTSD biological correlates identification

A recent review article has outlined the potential significance of using a social cognitive and affective neuroscience integrating approach in assessing complex PTSD (Lanius, Bluhm, & Frewen, 2011). The authors emphasized the importance of identifying deficits in emotional regulation and processing, self-awareness and self-reference, and their interconnections for the diagnosis and treatment of complex PTSD patients. In addition, complex PTSD poses specific treatment challenges, and there are many open questions regarding the therapeutic approach, treatment duration, and expected improvement (Cloitre et al., 2011). The characterization of complex PTSD biological correlates and the common or unique biological pathways involved in the pathogenesis of complex PTSD in comparison to PTSD would provide a better understanding of the disorder. This knowledge may in the end contribute to the identification of novel therapeutic strategies for complex PTSD.

## Conclusions and future research directions

1. Recent studies have examined and supported the diagnostic validity of complex PTSD as proposed for inclusion in ICD-11.
2. On the other hand, only a few studies have assessed the association of specific neurobiological correlates with complex PTSD. Independent replication and extension of pilot neuroimaging findings will be critical to identify brain circuits involved in the development of complex PTSD symptomatology.
3. Combinations of genetic and epigenetic studies are needed to address the role of genetic vulnerability, environmental factors, and their interaction in the development of complex PTSD symptomatology.
4. Characterization of autonomic and neurobiochemical correlates of complex PTSD and the overlap or potential differences with PTSD would help gain insight to the pathophysiology of the disorder and may aid in the identification of therapeutic targets.
5. Clearly defined and consistent subjects’ inclusion criteria would allow easier comparison of results between studies. In this respect, the introduction of complex PTSD as a diagnostic entity in ICD-11 would facilitate research efforts.
6. The potential role of the timing of the traumatic event and trauma type on biological findings associated with complex PTSD needs to be assessed, because initial studies have focused on complex PTSD related to childhood abuse.
7. A potential relationship between complex PTSD as proposed for ICD-11 and the dissociative PTSD subtype should be investigated.

## Supplementary Material

Biological correlates of complex posttraumatic stress disorder—state of research and future directions
