# Supplementary material for: Biological correlates of complex posttraumatic stress disorder—state of research and future directions
Source: Eur J Psychotraumatol. 2015 Apr 16;6:10.3402/ejpt.v6.25913. doi: 10.3402/ejpt.v6.25913 (PMC4401823; doi:10.3402/ejpt.v6.25913)
Supplement: Biological correlates of complex posttraumatic stress disorder—state of research and future directions [file EJPT-6-25913-s001.pdf]

Biologische Korrelate der komplexen posttraumatischen Belastungsstörung – Stand der Forschung und Ausblick

Zoya Marinova, Andreas Maercker

Die komplexe posttraumatische Belastungsstörung (komplexe PTBS) tritt mit dem Voll- oder subklinischen Bild der PTBS auf (Wiedererleben, Vermeidung, Übererregung) zu dem Symptome aus drei weiteren Gruppen kommen (Probleme der Emotionsregulation, negatives Selbstbild, Probleme mit Beziehungen). Komplexe PTBS ist für das ICD-11 als Diagnoseeinheit vorgeschlagen worden im Zusammenhang mit längerdauernden und komplexen Traumata. Wir beschreiben das heutige Wissen über die biologischen Korrelate der komplexen PTBS und vergleichen diese mit relevanten Befunden der PTBS. Während es inzwischen schon bestätigende Studien zur diagnostischen Validität der komplexen PTBS gibt, sind die Befunde zu deren biologischer Basis noch sehr begrenzt. Die ersten Bildgebungsstudien implizieren, dass es Überlappungen von PTBS und komplexer PTBS gibt, deuten aber darauf hin, dass möglicherweise zusätzliche Pathways einbezogen sind. Weitere Studien zu den biologischen Korrelaten der komplexen PTBS und eine Replikation bisheriger Befunde sind erforderlich, zu denen Bildgebung, neurobiochemische, genetische und epigenetische Ansätze gehören. Das Identifizieren von biologischen Pathways, die bei der komplexen PTBS verändert sind, ist wichtig für das Verständnis von deren Pathophysiologie und für verbesserte Therapiestrategien.

Citation: European Journal of Psychotraumatology 2015, **6**: 25913 - http://dx.doi.org/10.3402/ejpt.v6.25913
